# Imaging of Anterior Segment Tumours: A Comparison of Ultrasound Biomicroscopy Versus Anterior Segment Optical Coherence Tomography

**DOI:** 10.7759/cureus.52578

**Published:** 2024-01-19

**Authors:** Eleni Kottaridou, Adam Hatoum

**Affiliations:** 1 Accident and Emergency, Barts Health NHS Trust, London, GBR

**Keywords:** optical coherence tomography (oct), ubm, anterior segment ultrasound biomicroscopy, anterior segment optical coherence tomography (asoct), ultrasound biomicroscopy

## Abstract

Anterior segment tumours of the eye are relatively rare but can pose significant morbidity and mortality. We conducted a literature review to compare the performance of ultrasound biomicroscopy to anterior segment optical coherence tomography in the imaging of these tumours. A total of seven studies were included accounting for a cumulative 1,114 eyes. Ultrasound biomicroscopy has traditionally formed, and remains, the mainstay of tumour imaging due to its ability to penetrate pigmented lesions and delineate the posterior border of tumours, and the current evidence supports this.

## Introduction and background

Ocular tumours are relatively rare, accounting for fewer than 1% of all new cancer cases within the United States in 2023 [1]. Anterior segment tumours of the eye comprise only a proportion of these but can range from being entirely benign to signifying metastatic disease. Hence, their appropriate imaging and characterisation are crucial. These tumours can originate from any structure of the anterior segment, but most commonly from the iris and ciliary body. The majority are benign iridociliary cysts; however, even these can become large enough to cause compression and dislocation of the iris [2]. The most common primary malignancy of the anterior segment is malignant melanoma [3].

Management for these tumours can range from local (e.g. iridectomy, sector iridectomy, lamellar sclero-uvectomy) to radical resection (e.g. full-thickness eyewall resection), as well as ophthalmic radiation therapy [4]. Enucleation remains an option in uncontrollable secondary glaucoma in which all other treatments have failed [5].

Imaging plays an indispensable role in the diagnosis, surgical planning, and radiation treatment of these tumours. Ultrasound biomicroscopy (UBM) has traditionally been the gold standard; however, it comes with the disadvantages of requiring a highly skilled sonographer, being relatively time-consuming, and requiring water bath immersion of the eye, making it uncomfortable for the patient. More recently, newer imaging technologies, such as anterior segment optical coherence tomography (AS-OCT), have emerged, offering the attractive prospect of non-contact cross-sectional anterior segment imaging.

AS-OCT is significantly quicker, easier to use, and preferable to the patient. Furthermore, AS-OCT offers more compatibility with other technologies, such as artificial intelligence (AI), which are becoming more prevalent in wider medical practice and can assist clinicians greatly. AS-OCT images have already been used in the AI assessment of ocular disease; however, studies have typically been limited to the assessment of angle closure [6,7]. This potential of AS-OCT over UBM may represent a reason for its continued development.

History of UBM and AS-OCT

Since the advent of UBM in the 1990s by a group from the University of Toronto [8-11], it has largely formed the gold standard for the assessment of anterior segment tumours. UBM constituted the first method allowing subsurface imaging of the eye at a microscopic resolution. In modern practice, it has an important role in various fields such as corneal pathology, glaucoma, anterior segment surgery, and anterior segment tumours. Within the realm of anterior segment tumours, the main aim of UBM is the characterisation, diagnosis, and localisation of lesions, followed by surveillance and prognostication.

UBM uses high-frequency ultrasonography in the range of 20-50 MHz. In the 50 MHz mode, it provides a resolution of 25 µm, with a penetration of 5-6 mm [12]. This makes it a highly effective imaging modality for anterior segment tumours.

Since its inception in 1991, optical coherence tomography (OCT) has become widely used [13]. An imaging technique based on the reflectance of tissue using near-infrared (IR) light, rather than sound used in UBM, OCT is capable of producing high-quality cross-sectional images. Currently, the widespread use of OCT in ophthalmology remains predominantly for cross-sectional imaging of the retina and posterior eye.

Specifically targeted AS-OCT was first described in 1994 [14] and became commercially available in 2001 [15]. The main differences between UBM and AS-OCT are highlighted in Table 1. AS-OCT utilises the 1,310 nm wavelength of light (near-IR) to obtain 256 A-scans in 125 ms for a low-resolution image, or 512 A-scans in 250 ms for a high-resolution image. This yields an overall resolution of approximately 18 µm axially and 60 µm laterally, with a depth penetration of 3-6 mm depending on the scan type [16]. Its application today is largely in the fields of corneal refractive surgery, corneal transplant surgery, keratoconus, and other ocular surface diseases including tumours.

**Table 1:** Comparison of UBM to AS-OCT. AS-OCT = anterior segment optical coherence tomography; UBM = ultrasound biomicroscopy

	UBM	AS-OCT
Imaging principle	High-frequency ultrasound with a transducer frequency of 35–100 MHz	Utilises near infra-red light waves at a wavelength of 1,310 nm
Axial resolution	25 µm	18 µm
Depth penetration	6 mm	6 mm
Time	Time-consuming and uncomfortable for the patient	Quick and comfortable for the patient
Technician dependencies	Requires immersion eye bath and a highly skilled sonographer	Non-contact, machine-driven imaging
Applications	Angle-closure glaucoma, ciliary body cysts, neoplasms, and angle trauma	Corneal refractive surgery, corneal transplant surgery, keratoconus, and glaucoma

Despite the increased ease of use of AS-OCT, UBM remains the current gold standard for the assessment of anterior segment tumours. It can characterise all forms of lesions, aiding an accurate diagnosis. Several studies have compared the performance of UBM to AS-OCT for glaucoma and anterior segment surgery [17,18]. However, currently, no comprehensive review exists to address the question of superior imaging modality. In this paper, we aim to compare the performance of UBM and AS-OCT in the imaging of anterior segment tumours. This review aims to discuss studies which have directly compared the performance and explore the relative advantages and disadvantages of each.

## Review

Methodology

A literature search through the PubMed database was performed by two separate researchers in November 2023. The study aimed to investigate the UBM and AS-OCT techniques in patients with anterior segment tumours. The following terms were used for the literature search: (((((ultrasound b-scan) OR (UBM)) OR (Ultrasound Biomicroscopy)) OR (biomicroscopy, ultrasound[MeSH Terms])) AND ((((((((((((ciliary body melanoma) OR (ciliary body nevus)) OR (ciliary body tumour)) OR (iris cyst)) OR (iris melanoma)) OR (iris nevus)) OR (iris tumour)) OR (Iris Neoplasm)) OR (anterior segment tumour)) OR (anterior segment tumour)) OR (iris tumour)) OR (ciliary body tumour))) AND (anterior segment optical coherence tomography). Full-text articles published in the English language were included in this study.

The study collected data on various characteristics and demographics of the patients. This included information on the type of study conducted and the number of eyes examined. Table 2 schematically illustrates these parameters. The studies analysed various types of anterior segment tumours, including cysts and pigmented or hypopigmented tumours.

**Table 2:** Study characteristics. AS-OCT = anterior segment optical coherence tomography; UBM = ultrasound biomicroscopy

	Pavlin et al. (2009) [16]		Bianciotto et al. (2011) [12]		Razzaq et al. (2011) [19]		Krema et al. (2013) [20]		Shields et al (2017) [21]		Kose et al. (2020) [22]		Konopinska et al. (2020) [23]	
City, country	Toronto, Canada		Pennsylvania, USA		Leiden, Netherlands		Toronto, Canada		Pennsylvania, USA		Ankara, Turkey		Bialystok, Poland	
Type of study	Observational study		Observational study		Observational study		Observational study		Observational study		Observational study		Observational study	
Number of patients	18		200		61		37		672		37		89	
Type of lesions	Five irido-ciliary cysts, four small hypopigmented iris tumours, three pigmented tris tumours, one large hypopigmented iris tumour, and five irido-ciliary tumours		96 iris stroma tumours, 44 iris pigment epithelium tumours, 32 iris and ciliary body tumours, 14 ciliary body tumours, four tumours in the sclera, two tumours in the ciliary body and choroid, and two tumours in the iris, ciliary body, and choroid		39 iris nevus and 22 iris and/or anterior ciliary body melanoma		37 non-pigmented iris tumours		49 cysts in the pupillary margin, 188 midzone cysts, 424 peripheral cysts, and 11 dislodged/free-floating cysts		35 primary cysts, 31 primary iris pigment epithelium cysts (pupillary 0, mid-zonal four, peripheral 26, dislodged/free-floating one), four primary stromal cysts (congenital one, acquired three), and two secondary stromal cysts (due to trauma/surgery two, fue to tumour 0, and parasitic 0)		37 cysts (26 solid iris tumours, seven ciliary body tumours, four corneal tumours)	
Number of patients based on diagnostic mean	UBM	AS-OCT	UBM	AS-OCT	UBM	AS-OCT	UBM	AS-OCT	UBM	AS-OCT	UBM	AS-OCT	UBM	AS-OCT
	18	18	200	200	49	46	37	37	672	672	26	4	74	13
Conclusions	UBM and AS-OCT are equal in anterior surface and hypopigmented tumours. UBM is superior over AS-OCT in posterior surface and hyperpigmented tumours		UBM is equal to AS-OCT in visualising the anterior margin of the tumour. UBM is better for the visualisation of the posterior margin. UBM has superior image quality		Similar outcomes for both UBM and AS-OCT for iris nevi. UBM is superior to AS-OCT for iris or ciliary body melanoma. Similar thickness measurements with both techniques. UBM is superior in detecting tumours extending into the ciliary body		UBM and AS-OCT are equal in detecting the anterior margin of the tumour. AS-OCT detects surface irregularities. UBM is better at visualising posterior margins. UBM is more accurate in detecting tumour thickness. AS-OCT is affected by optical aberrations		UBM is superior in visualising both anterior and posterior margins of the cyst		UBM distinguishes cysts from solid tumours. UBM is superior in identifying tumour thickness and diameter. AS-OCT is incapable of visualising the posterior surface of the tumour. AS-OCT is affected by the levels of pigmentation		Sequential UBMs detect changes in tumour size	

Results

**Figure 1:**
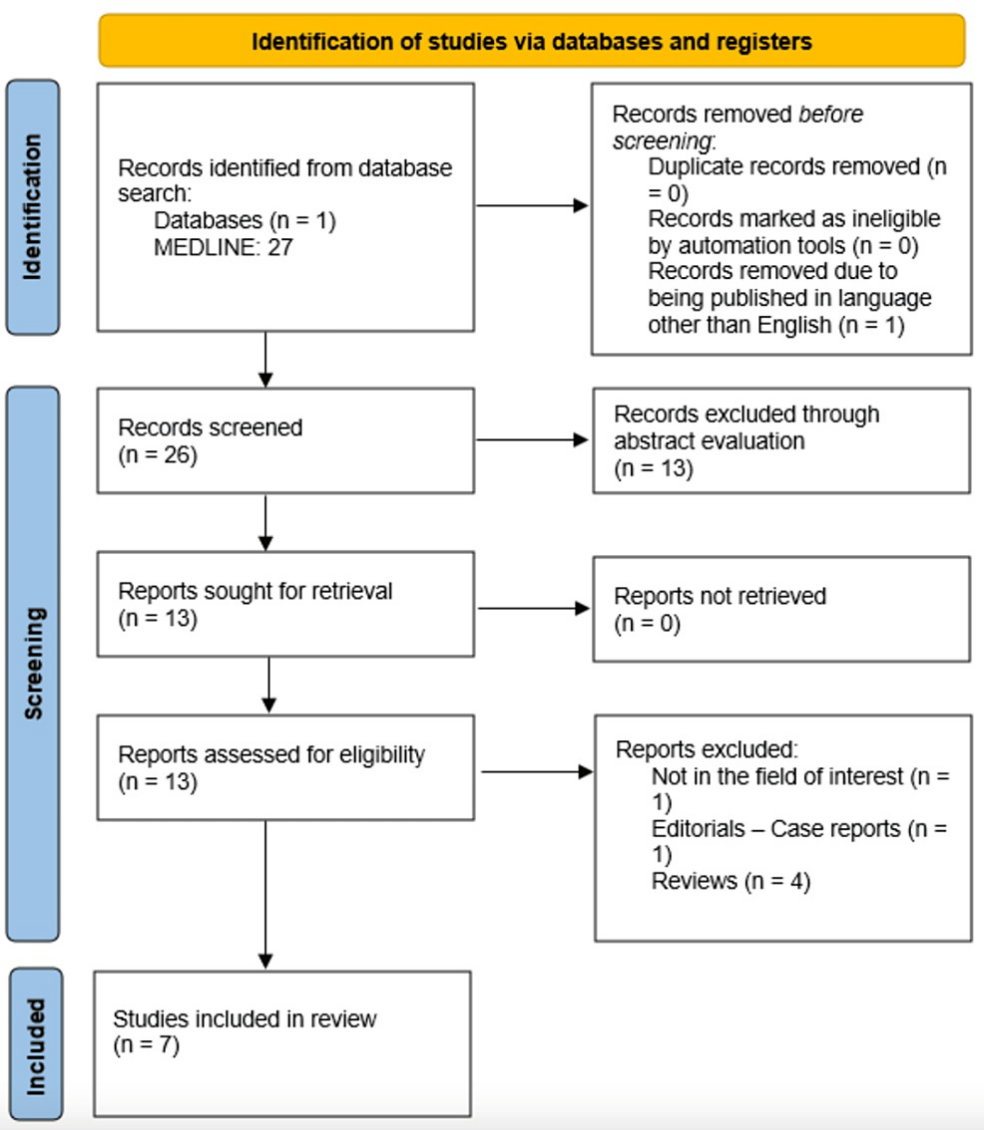
Preferred Reporting Items for Systematic Reviews and Meta-Analyses flowchart.

This literature review serves as a comprehensive synthesis of the existing information pertaining to the utilisation of UBM in contrast to AS-OCT among patients diagnosed with anterior segment tumours, drawing upon insights from seven observational studies. Initially, a search of the literature identified 27 studies, as shown in Figure 1. However, 19 studies were excluded due to their lack of relevance to the primary aim of this review, and one study was omitted as it was not published in the English language. All studies ultimately included in the final review adhered to the predetermined inclusion criteria, accounting for a total of 1,114 eyes.

The initial study by Pavlin et al. in 2009 [16] examined 18 patients with varying characteristics of anterior segment tumours. All patients underwent examinations utilising both UBM and AS-OCT. These patients were categorised into five distinct groups based on tumour location, size, and pigmentation level. In cases of irido-ciliary cysts observed in five patients, AS-OCT struggled to visualise the thin cyst wall, the underlying lens, zonules, and ciliary processes. However, it successfully outlined the borders of thick-walled cysts, particularly in hypopigmented irises. In contrast, UBM emerged as a comprehensive imaging technique that delineated the cyst walls and surrounding structures effectively. Regarding four small hypopigmented iris tumours, both UBM and AS-OCT demonstrated comparable success in penetrating the walls, offering moderate internal reflectivity, and providing adequate thickness measurements. However, in the case of a large hypopigmented iris tumour, AS-OCT exhibited limitations in reaching the tumour’s deeper parts and identifying its posterior borders. UBM imaging surpassed AS-OCT in three patients with pigmented iris tumours and five patients with irido-ciliary tumours. The ability of AS-OCT to penetrate tissues is compromised in the presence of pigmented tumour cells due to their high reflectivity, which impedes precise measurements. Conversely, UBM can penetrate the entire thickness of the lesion, overcoming this limitation.

In a study by Bianciotto et al. in 2011 [12], an evaluation was conducted among 200 patients with anterior segment tumours situated in various locations. Both UBM and AS-OCT were used for assessment. The comparative analysis focused on aspects such as overall tumour visualisation; resolution of internal tumour structures; visibility concerning the anterior, posterior, and lateral margins; presence of posterior tumour shadowing; and image resolution in relation to the level of pigmentation. The findings indicated that while both techniques were equally effective in visualising the anterior margin (90% UBM vs. 82% AS-OCT), UBM notably provided a significantly clearer view of the posterior margin (90% UBM vs. 29% AS-OCT). Furthermore, the percentage of tumour shadowing was significantly lower with UBM (5%) compared to AS-OCT (72%). Overall image quality was also notably superior with UBM (80%) in contrast to AS-OCT (68%).

In the same year, a subsequent study conducted by Razzaq et al. [19] examined 61 patients diagnosed with melanocytic iris tumours. Among them, 17 patients underwent assessment with Pentacam, 12 with SL-OCT, and 46 with AS-OCT. The findings of 49 patients were compared to UBM. The research team developed an ordinal data classification system based on five parameters, namely, overall tumour visibility, structural characteristics, dimensions, precise location, expansion in the anterior chamber angle, and ciliary body visibility. For iris nevi, 28 patients showed similar outcomes with UBM and AS-OCT regarding the aforementioned parameters. The average tumour thickness measured 0.86 mm in both techniques (p = 0.65; 95% confidence interval (CI) = -0.0075 to 0.0048). However, in cases of iris or ciliary body melanoma, AS-OCT demonstrated similarity with UBM in eight out of 14 patients. The mean thickness remained comparable, registering at 2.04 mm with UBM and 2.01 mm with AS-OCT (p = 0.11; 95% CI = -0.0063 to 0.0520). Furthermore, the study highlighted UBM’s superiority in detecting tumours that extend into the ciliary body.

In 2013, Krema et al. [20] conducted a retrospective analysis involving 37 patients diagnosed with non-pigmented iris tumours that did not extend to the ciliary body. These patients underwent both UBM and AS-OCT examinations. The study aimed to assess the visibility of the anterior and posterior surfaces, visualise internal tumour structures, and measure tumour thickness. It also considered the presence of image artefacts or optical aberrations that might compromise the overall image quality. The study findings revealed that although both techniques yielded high-resolution images of the anterior surface in 100% of the tumours, AS-OCT was capable of detecting surface irregularities that were not apparent with UBM, notably tapioca-like and irregular surface tumours which showed finger-like projections from the tumour surface. Conversely, UBM demonstrated significantly superior imaging of the posterior surfaces of the tumours, while AS-OCT failed to yield results in 14% of the cases. In 24% of the tumours, both techniques exhibited limited heterogeneity in internal structures, whereas UBM successfully measured tumour thickness in all patients compared to 86% of patients with AS-OCT. Notably, the median thickness difference between UBM and AS-OCT in 32 tumours was 0.085 mm. AS-OCT was found to offer a narrower field of view, restricting complete tumour visualisation along its lateral boundaries. Additionally, it was more susceptible to optical aberrations and image artefacts than UBM, leading to mirror-image reflections.

In 2017, Shields et al. [21] conducted compared UBM and AS-OCT in a cohort of 672 patients diagnosed with iris pigment epithelium cysts. Following the classification of primary cysts based on their location, the team detailed the distinct characteristics and findings associated with each type of cyst. Notably, the team emphasised the advantage of UBM in visualising both the anterior and posterior cyst walls with lower resolution, contrasting with AS-OCT, which primarily showcases the anterior wall of the cyst using higher resolution.

Likewise, in 2020, an examination was conducted among 37 patients diagnosed with iris cysts, by Kose et al [22], among whom 26 patients underwent UBM examinations, while four patients underwent AS SS-OCT assessments. The primary focus of the study involved analysing the clinical and demographic attributes of these 37 patients based on the localization of their iris cysts. However, the researchers highlighted the significance of utilising UBM to distinguish cysts from solid tumours. Cysts typically manifest as hollow, thin-walled lesions, contrasting with solid tumours that exhibit solid inner structures. Additionally, UBM’s capacity for high penetration enables the identification of tumour thickness and diameter, regardless of pigmentation levels, and facilitates the visualisation of tumour extension to the ciliary body. In contrast, AS-OCT lacks the ability to display the posterior surface of the tumour, thereby diminishing its relevance in imaging iris cysts, particularly in cases involving pigmentation.

Finally, a second retrospective analysis was published in the same year by Konopinska et al. [23], focusing on the prolonged monitoring of 89 patients suspected of having anterior segment tumours. These patients underwent continuous evaluation based on their UBM findings, particularly the assessment of a 20% or more increase in the lesion’s height in comparison to prior measurements across two separate tests. As part of the diagnostic confirmation, 13 (14.6%) patients were subsequently examined using AS-OCT. The study findings highlighted that the most prevalent anterior segment tumours observed were cysts (37.42%) and solid iris tumours (33.37%). UBM successfully identified 74 (80.1%) cases, while AS-OCT recognized five cases of iris nevus and two cases of corneal leukoma. The researchers underscored the significance of sequential UBM examinations to detect alterations in tumour size, a factor evident in only 2.2% of patients within this study. Furthermore, the study delved into various demographic characteristics of patients with anterior segment tumours, including age, gender, best-corrected visual acuity, and intraocular pressure. 

Discussion

In accordance with the aforementioned findings, UBM and AS-OCT fulfil distinct purposes based on the specific investigative requirements for various types of anterior segment tumours.

Imaging of the Anterior Tumour Surface

Both UBM and AS-OCT yielded high-resolution results [11,15,18-22], enabling comprehensive visualisation of the anterior borders of anterior segment tumours. It is crucial to emphasise that AS-OCT, in particular, excels in providing high-resolution images owing to its ability to analyse images with greater detail [11]. AS-OCT demonstrated superiority over UBM in detecting irregular surface tumours [19]; however, additional investigations are warranted for a thorough assessment.

Imaging of the Posterior Tumour Surface

AS-OCT presented images of lower resolution because of its inability to fully reflect light and penetrate tumours with a thicker consistency. This limitation can also be ascribed to the occurrence of tumour shadowing, a phenomenon rarely observed with UBM [11,15,18,19,21]. These findings are particularly accentuated in instances of hyperpigmented lesions, such as ciliary body cysts or tumours of the iris pigment epithelium [11,15,18-22].

Thickness of the Tumour

UBM demonstrated superiority in measuring the thickness of anterior segment tumours by providing accurate measurements for tumours of varying thickness. In contrast, AS-OCT induces a gradual attenuation of light reflected from the anterior to the posterior surface of the tumour, hindering precise measurements in thicker tumours. It is noteworthy that disparate thickness values, as measured by both UBM and AS-OCT, have been reported, confirming the superiority of UBM over AS-OCT [15,18,19,22]. Nevertheless, it is imperative to establish a specific threshold for accurate AS-OCT usage in lieu of UBM.

Image Quality

Various research endeavours have highlighted the superiority of UBM over AS-OCT concerning the comprehensive visualisation of anterior segment tumours. Specifically, AS-OCT is susceptible to optical aberrations, resulting in mirror-image reflections stemming from diverse optical sources or densities, such as the presence of intraocular lenses, ectropion uvea, or dense corneas [19]. UBM appears to be less prone to optical aberrations as it employs sound waves for image capture instead of light, facilitating a less intricate clinical examination [19]. Furthermore, UBM offers enhanced visualisation of ciliary body tumours, providing a thorough assessment of tumour borders. This renders UBM particularly suitable for investigations when the extension of an iris tumour is suspected [11,19,21]. Nevertheless, recent studies have underscored the ability of AS-OCT to delineate the internal vascular structure of anterior segment tumours [21].

## Conclusions

AS-OCT represents an advancement in anterior segment imaging which, as explained, has found many uses in various fields of ophthalmology. However, UBM remains the better imaging technique for anterior segment tumours, predominantly due to its ability to penetrate pigmented lesions and delineate the posterior margin of tumours. The only areas in which AS-OCT can match UBM are imaging of non-pigmented lesions and anterior tumour margin delineation. Until AS-OCT can accurately visualise all forms of anterior segment tumours, UBM will likely remain the imaging modality of choice.
